# Clinical Parameters and Gut Microbiome Changes Before and After Surgery in Thoracic Aortic Dissection in Patients with Gastrointestinal Complications

**DOI:** 10.1038/s41598-017-15079-0

**Published:** 2017-11-09

**Authors:** Shuai Zheng, Shulin Shao, Zhiyu Qiao, Xue Chen, Chunmei Piao, Ying Yu, Feng Gao, Jie Zhang, Jie Du

**Affiliations:** 1 Beijing Anzhen Hospital, Capital Medical University, Beijing, 100029 China; 2 Beijing Collaborative Innovation Centre for Cardiovascular Disorders, Beijing, 100029 China; 3 The Key Laboratory of Remodeling-Related Cardiovascular Diseases, Ministry of Education, Beijing Institute of Heart Lung and Blood Vessel Diseases, Beijing, 100029 China; 4 Department of Gastroenterology, Beijing Anzhen Hospital, Capital Medical University, Beijing, 100029 China; 5 Department of Cardiovascular Surgery, Beijing Aortic Disease Centre, Beijing Anzhen Hospital, Capital Medical University, Beijing Institute of Heart Lung and Blood Vessel Diseases, Beijing Engineering Research Centre for Vascular Prostheses, Beijing, 100029 China

## Abstract

Thoracic aortic dissection (TAAD) is one of the most common types of aortic diseases. Although surgery remains the main method of treatment, the high rate of postoperative gastrointestinal complications significantly influences the effects of surgery and the recovery process. Moreover, the mechanisms underlying this disease remain unclear. To address these problems, we examined changes in the gut microbiota in 40 thoracic aortic dissection patients with abdominal complications after surgery. Levels of white blood cells (WBC), neutrophile granulocytes (NE), alanine aminotransferase (ALT), and aspartate aminotransferase (AST) were higher in all patients after surgery. Levels of inflammatory cytokines, including interleukin (IL)-2, IL-6, IL-8, and IL-10, were also higher after surgery. A metagenome analysis revealed that levels of *Oscillibacter*, *Anaerotruncus*, *Alistipes*, and *Clostridium difficile* were higher after the operation. The abundance of functional genes, such as the spermidine/putrescine transport system permease protein, the flagellar motor switch protein, and branched-chain amino acid transport system proteins, was also higher post-surgery. These changes likely contribute to diarrhea, bloating, gastrointestinal bleeding, and other abdominal complications after surgery, and our research opens up new treatment possibilities for patients suffering from abdominal complications after surgical treatment.

## Introduction

Aortic dissection (AD) is one of the most common types of aortic disease. The prevalence of AD is about 2 to 16 cases/100,000 inhabitants/year^1^, although such incidence is not high, its outcome is frequently fatal: when the interlining bursts, the mortality rate reaches 100%.

Surgical intervention is suggested for treating Stanford Type A aortic dissection^2^, and is still the preferred method for such kind of AD^3^, and anti-hypertensive treatment should begin at the occurrence of systemic hypertension. However, postoperative complications are frequently encountered, which impact the prognosis and increase the treatment costs. It is estimated that, for patients with AD, the operative mortality rate ranges from 5% to 10% and may reach 70% in cases with complications^4^ after surgery. One of the more serious complications after aortic surgery is gastrointestinal complications (GICs)^5^. GICs are not rare in aortic-related surgeries, occurring in approximately 2–50% of open cardiac operations, neurosurgical operations, descending thoracic or thoracoabdominal aortic repairs, and cardiac or lung transplantations^5–9^. In Anzhen Hospital, more than 1200 AD surgeries were performed from 2008 to 2016. In follow-up, 70–80% of postoperative patients were found to have GICs such as diarrhea, abdominal distention, difficulty defecating, gastrointestinal bleeding, and other digestive system complications (unpublished data). However, the causative factors and underlying mechanisms remain unclear.

The human gut intestinal flora is closely related to human health. Gut microbiota dysbiosis is involved in the occurrence and development of various diseases, including coronary heart disease, hypertension, diabetes, inflammatory bowel diseases, and others^10–13^. Specifically, many abdominal symptoms, such as abdominal pain, diarrhea, and abdominal distension, are closely related to intestinal flora^14^. Moreover, gut bacteria may be the origin of postoperative sepsis and multiple organ dysfunction syndrome (MODS). Conversely, gut microbiota may be disturbed by stressful conditions such as surgery. Thus, gut microbiota should be taken as a sign and treatment target for complications after AD surgery. However, there are no reports on changes in gut microbiota in AD patients.

We characterized changes in the gut microbiome in AD patients with GICs after surgery using high-throughput sequencing. Gut microbiota composition, metagenome changes, and related metabolic pathway changes were characterized. We also evaluated the systemic inflammatory response in postoperative patients and determined its correlation with changes in the microbiota. Our results provide the first glimpse of the dysbiosis of the gut microbiota in AD patients with surgical GICs.

## Results

### A systemic inflammatory response after surgery

**Figure 1:**
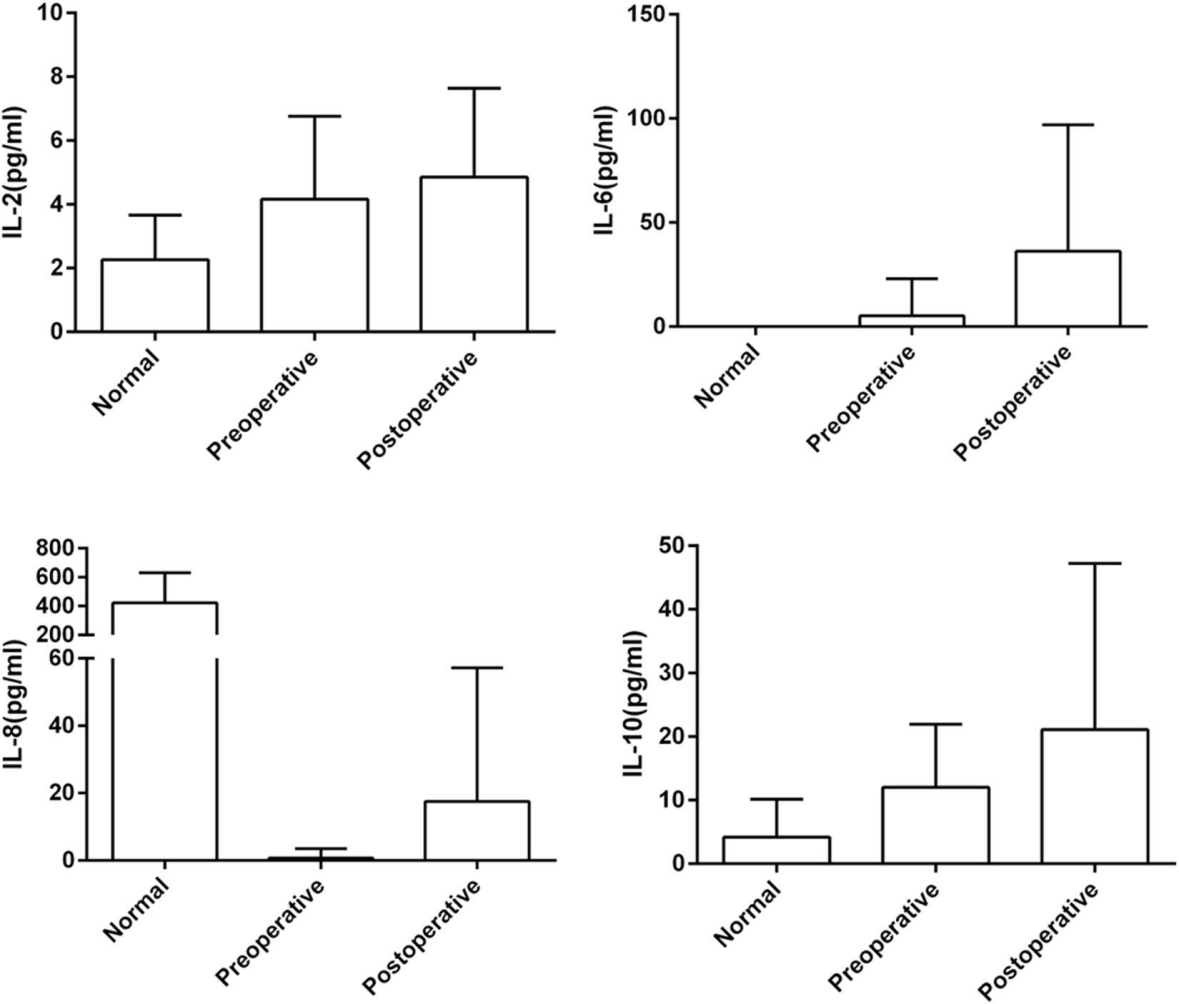
Inflammatory cytokines changes before and after surgery. Plasma IL-2, IL-6, IL-8, and IL-10 concentrations (pg/ml) were tested by ELISA respectively. The average levels of each group for each cytokine were as shown in the bar graphs, and groups were as labeled under X axis.

WBC (white blood cells), NE (neutrophile granulocytes), AST (aspartate aminotransferase), and ALT (alanine aminotransferase) levels were significantly higher in plasma after surgery (Table 1), indicating systemic inflammation as well as liver injury. ELISAs on blood samples revealed that inflammatory cytokines, including IL-2, IL-6, and IL-10, had a tendency to increase after surgery, although the difference was not significant (Fig. 1). Such results were in accordance with the WBC and NE levels. By contrast, IL-8 was the highest in healthy volunteers and was significantly lower in the plasma of thoracic ADs patients both before and after surgery (it was slightly higher after surgery). Besides, the average level of serum creatinine (sCr) raised from 97.68 ± 43.17 μmol/L (mean ± SD) before surgery to 123.23 ± 43.42 μmol/L after surgery. Specifically, there were 16 patients whose post-surgery sCr was above the normal level (57–111 μmol/L), and 7 of them had their pre-surgery sCr higher than the normal level, suggesting acute kidney injury in the perioperative period. The other 24 patients showed normal sCr values during the perioperative period. And none of 40 patients had infection in the perioperative period.

**Table 1:** Cycling inflammatory cell concentrations and liver function index.

Items	Preoperative N = 30	Postoperative N = 30	Independent-Samples T Test
**WBC**	8.47 ± 3.88	14.60 ± 4.16	*P* < 0.001
**NE**	6.36 ± 3.86	12.19 ± 3.72	*P* < 0.001
**ALT**	24.83 ± 18.38	56.90 ± 67.04	*P* < 0.001
**AST**	24.07 ± 11.06	47.37 ± 30.94	*P* < 0.001

White blood cells and neutrophils in blood samples were detected, and their concentration (gram per liter blood) were significantly increased post-surgery. Also the two liver function index, alanine aminotransferase (ALT) and aspartate amino transferase (AST) were significantly increased post-surgery (concentration as U/L). Data are shown as mean ± SD, values were compared by Student’s t-test, and p values were as in the table.

### Gut microbiota diversity changed after surgery

**Figure 2:**
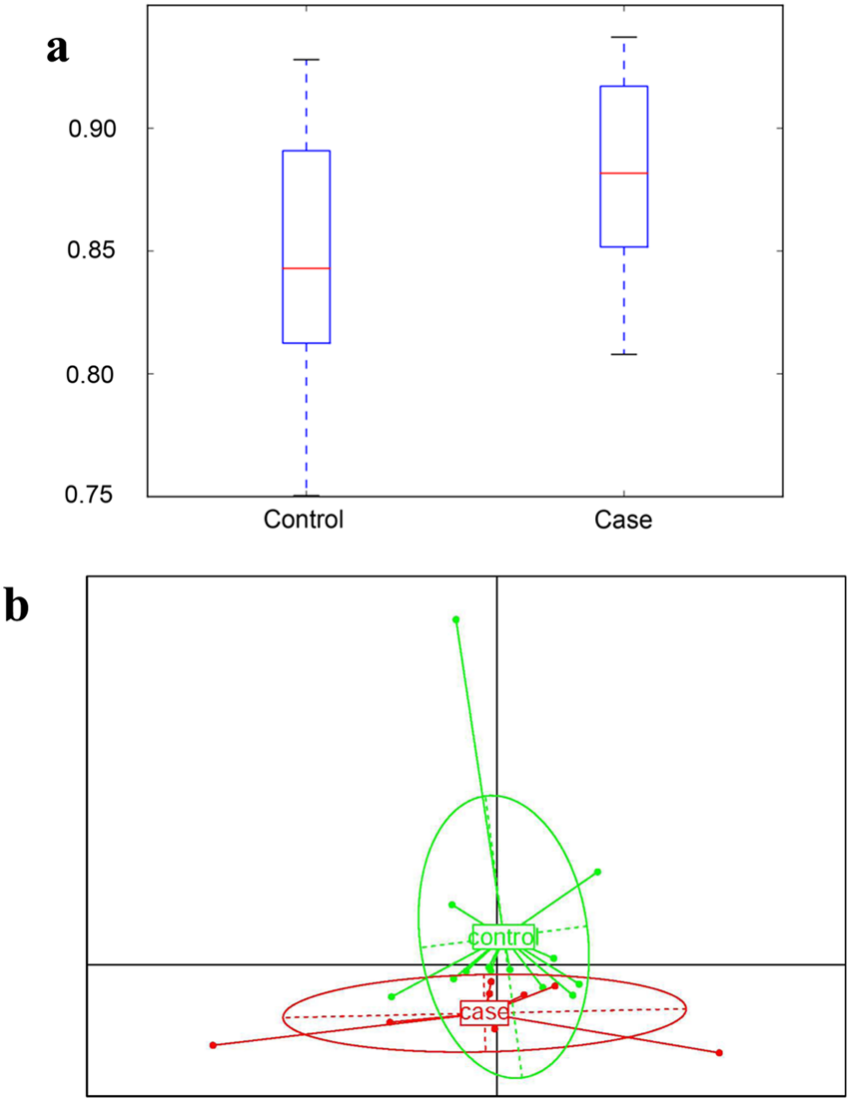
Diversity analysis of gut microbiota changes before and after surgery. (**a**) Alpha-diversity analysis of Simpson’s test. The value of each group was shown in the box plot. (**b**) Beta-diversity analysis was shown in the PCA graph, the green cycle and “control” represented the pre-surgery group, and the red cycle and “case” represented the post-surgery group.

Next, we examined changes in the gut microbiota after surgery to determine whether the gut microbiota is related to the systemic inflammatory status. We first evaluated the changes in gut microbiota diversity. Alpha diversity (by Simpson’s test) showed slight changes in the gut microbiota before and after surgery (Fig. 2a), suggesting that surgery had little influence on richness of taxonomy and number in the gut flora (104 species and 100 genera, respectively, before and after surgery in patient stool samples). We also compared gut flora changes using beta diversity. As shown in Fig. 2b, the microbial compositions differed strikingly between the two groups before and after surgery.

### Microbial abundance was greatly changed after surgery

**Figure 3:**
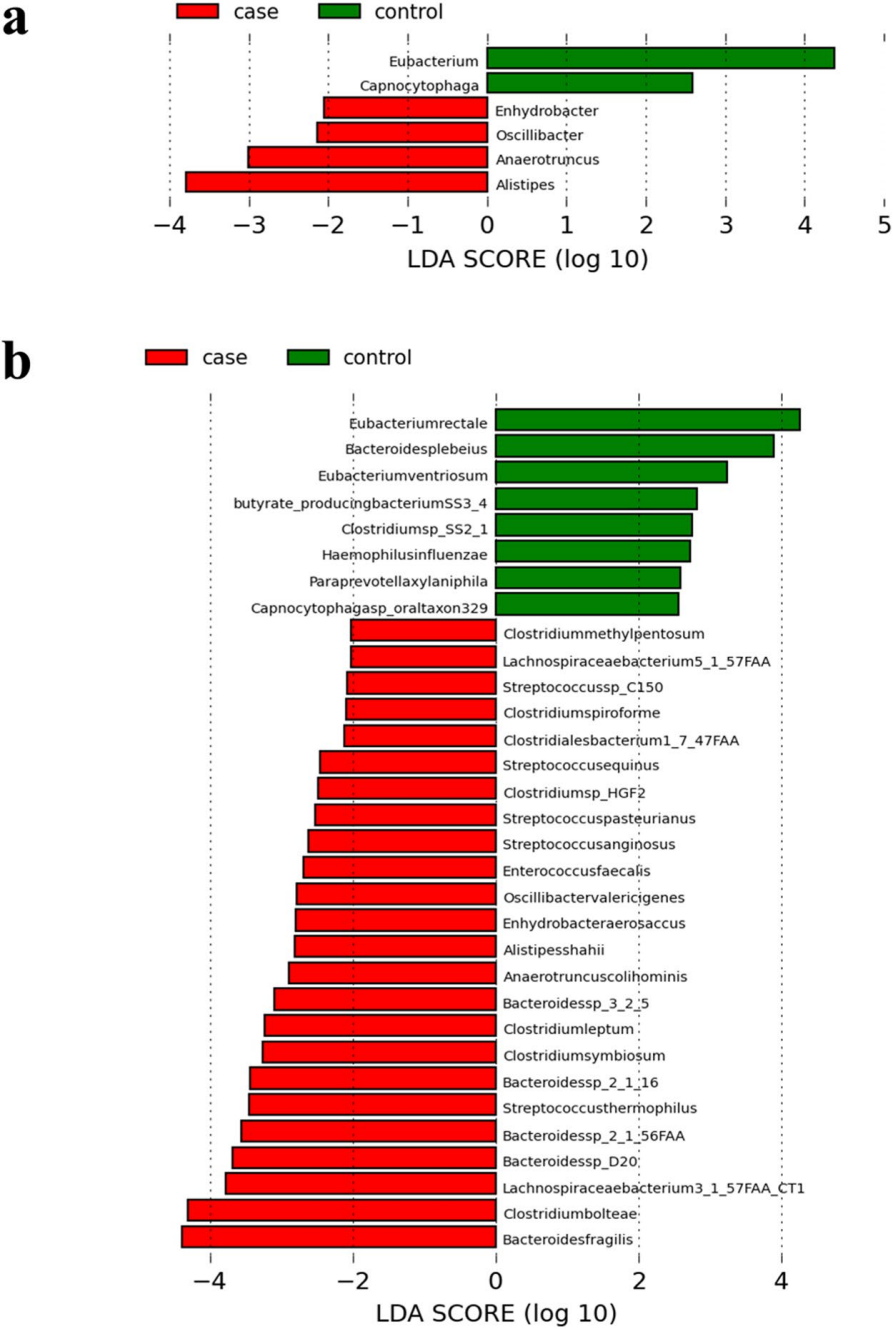
Abundant changes of gut flora before and after surgery. Histogram of the linear discriminant analysis (LDA) scores for significantly changed genera (3a) and species (3b) bacteria. The related bacteria names of each column were listed at the bottom, and the score number was shown on the X axis. The red bars and “case” represented bacteria which were increased after surgery, while the green bars and “control” represented bacteria which were decreased after surgery.

Because beta diversity showed that the microbial composition changes after surgery, we further investigated the changes in microbial abundance. At the genus level, *Enhydrobacter*, *Oscillibacter*, *Anaerotruncus*, and *Alistipes* were significantly higher after surgery, while *Eubacterium* and *Capnocytophaga* were significantly decreased after surgery (Fig. 3a). At the species level, the gut microbiota after surgery was enriched in *Bacteroides fragilis*, *Clostridium bolteae* and *Lachnospiraceae bacterium*, while gut microbiota before surgery was enriched in *Enbacterium rectale*, butyrate-producing bacteria, and *Bacteroides plebeius*, among others (Fig. 3b).

### The abundance of functional genes and related metabolic pathways was changed after surgery

In addition to changes in bacterial abundance, we also found that the abundance of various genes in the metagenome differed significantly before and after surgery. The intestinal flora displayed higher abundance of genes involving generation of spermidine/putrescine transport system permease proteins, flagellar motor switch proteins, and branched-chain amino acid transport system proteins in postoperative patients (Table 2, upper part), but displayed a lower abundance of genes involving generation of periplasmic nitrate reductase, cathepsin, nitroreductase, nitrite reductase, tryptophan synthase, and sulfur carrier proteins (Table 2, lower part). We further investigated potential changes in the metabolic pathways in which these significantly altered genes are involved. The abundance of tryptophan biosynthesis, competence-related DNA transformation transporter and tetracycline resistance proteins decreased after surgery, while protein pathways involved in cysteine biosynthesis were significantly enhanced after surgery (Table 3).

**Table 2:** Abundant changes of genes within metagenome.

Class	Function	Tendency after surgery
**K01997**	branched-chain aminoacid transport system permease protein	rise
**K11071**	spermidine/putrescine transport system permease protein	rise
**K12960**	5-methylthioadenosine/S-adenosyl homocysteine deaminase	rise
**K01996**	branched-chain amino acid transport system ATP-binding protein	rise
**K02408**	flagellar hook-basal body complex prote	rise
**K02406**	flagellin	rise
**K02417**	flagellar motor switch protein	rise
**K07684**	NarL family, nitrate/nitrite response regulator NarL	rise
**K02567**	periplasmic nitrate reductase NapA	decline
**K01368**	CTSS; cathepsin S	decline
**K11075**	putrescine transport system permease protein	decline
**K10678**	nitroreductase	decline
**K00772**	5′-methylthioadenosine phosphorylase	decline
**K13747**	carboxynorspermidine decarboxylase	decline
**K01609**	indole-3-glycerol phosphate synthase	decline
**K17877**	nitrite reductase (NAD(P)H)	decline
**K00767**	nicotinate-nucleotide pyrophosphorylase	decline
**K01695**	tryptophan synthase alpha chain	decline
**K01696**	tryptophan synthase beta chain	decline
**K03154**	sulfur carrier protein	decline
**K05366**	penicillin-binding protein 1A	decline

Functional genes which had abundance greatly changed after surgery were in the table. Each gene had its KEGG ID and the functional description listed, and the changing trend after surgery were followed. Upper part were genes with abundant increase after surgery, and lower part were genes with abundant decrease after surgery. All the significances of abundant differences were decided by the Student’s t test.

**Table 3:** Abundant changes of relative pathways within metagenome.

Module	Function	Tendency after surgery
**M00667**	Tetracycline resistance, efflux pump	decline
**M00060**	Lipopolysaccharide biosynthesis	decline
**M00704**	Tetracycline resistance	decline
**M00122**	Cobalamin biosynthesis	decline
**M00338**	Cysteine biosynthesis,homocysteine + serine = >cysteine	rise

Functional pathways which had abundance greatly changed after surgery were in the table. Each pathway had its KEGG ID and the functional description listed, and the changing trend after surgery were followed. All the significances of abundant differences were decided by the Student’s t test.

### Changes in functional genes and metabolic pathways are correlated with changes in bacterial composition

**Figure 4:**
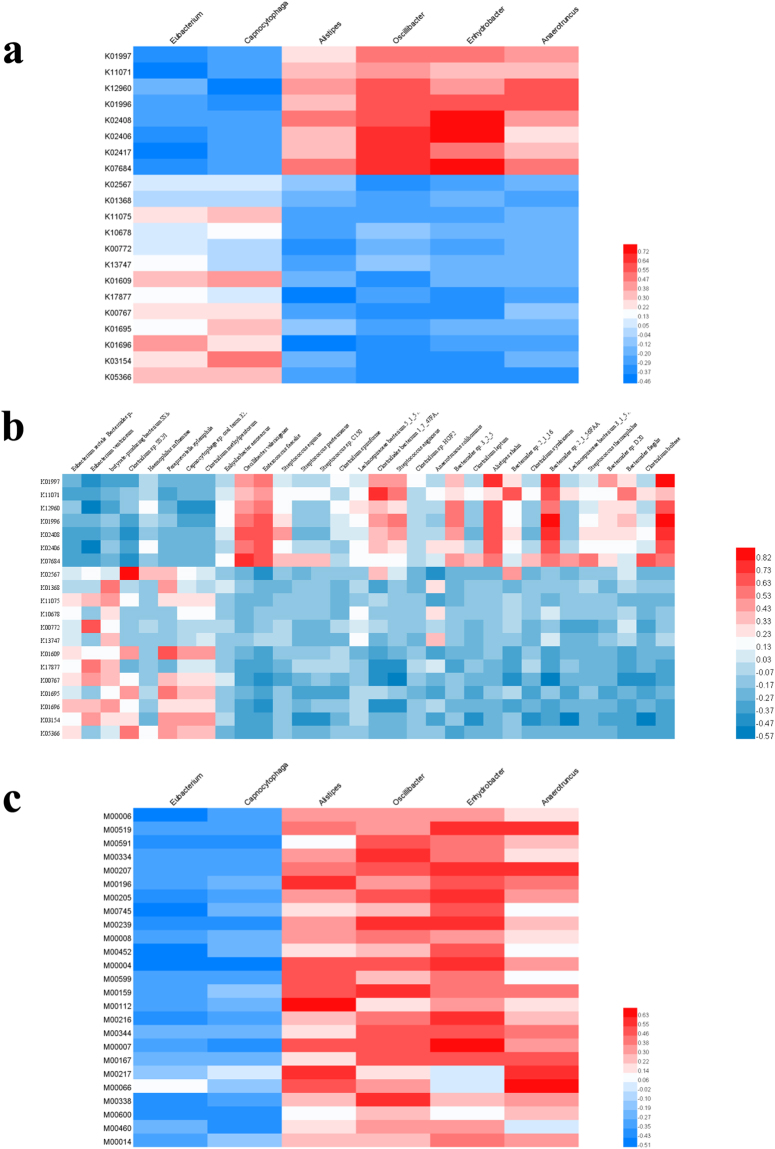
Correlation between significant changed bacteria and functional genes and pathways. (**a**) Heatmap of correlation between significant changed genera and functional genes. KEGG ID of each gene was listed on the left side, and the genera names were listed on the top. (**b**) Heatmap of correlation between significant changed species and functional genes. KEGG ID of each gene was listed on the left side, and the species names were listed on the top. (**c**) Heatmap of correlation between significant changed genera and functional pathways. KEGG ID of each pathway was listed on the left side, and the genera names were listed on the top. For all the graphs, the intensity of correlation were shown in different colors, as indicated by the symbol graphs, and the results were clustered by rows and columns respectively.

To determine whether the changes in functional genes and pathways were caused by variation in intestinal microbiota structure, we analyzed the correlations between functional genes/pathways and microbiota species. At the genus level, the abundant upregulated genes were all positively correlated with bacterial strains whose abundance was higher after surgery, and the abundant downregulated genes were all negatively correlated with those strains. The same was true for altered genes and bacterial strains whose abundance was lower after surgery (Fig. 4a) (all p < 0.05 and |r| > 0.3). However, at the species level, bacterial species whose abundance was higher after surgery were positively correlated with approximately half of the abundant upregulated genes post-surgery (but negatively correlated with all abundant downregulated genes), and only approximately 2/3 of the bacterial species whose levels were lower after surgery were positively correlated with abundant downregulated genes (all species were negatively correlated with all abundant upregulated genes post-surgery). Similar results were obtained for the correlations between functional pathways and gut flora (i.e., abundant upregulated pathways were all positively correlated with species whose levels were higher after surgery but negatively correlated with all species whose levels were lower) (Fig. 4c). Moreover, the cysteine biosynthesis pathway was correlated with changes in microbial structure after surgery (Tables 4,5).

**Table 4:** Correlation between significant changed genera and functional pathway.

Genus	M00338
**Eubacterium**	−0.385137826
**Capnocytophaga**	−0.335719393
**Alistipes**	0.271114543
**Oscillibacter**	0.592058811
**Enhydrobacter**	0.290050709

The significantly increased pathway (M00338) post-surgery and its correlation with significantly changed bacteria at genus level. The KEGG ID and bacterial names were listed, and the r value of Spearman correlation tests were as shown.

**Table 5:** Correlation between significant changed species and functional pathway.

Species	M00338
**Eubacterium rectale**	−0.320729622
**Bacteroides plebeius**	−0.322996206
**Eubacterium ventriosum**	−0.21741808
**butyrate-producing bacterium SS3/4**	−0.482027006
**Clostridium sp. SS2/1**	−0.096419179
**Haemophilus influenzae**	−0.202402005
**Paraprevotella xylaniphila**	−0.32964937
**Capnocytophaga sp. oral taxon 329**	−0.335712812
**Clostridium methylpentosum**	0.047937319
**Enhydrobacter aerosaccus**	0.289571189
**Oscillibacter valericigenes**	0.618160706
**Enterococcus faecalis**	0.04801544
**Streptococcus equinus**	0.074151663
**Streptococcus pasteurianus**	0.074803317
**Streptococcus sp. C150**	−0.108000522
**Clostridium spiroforme**	0.28868248
**Lachnospiraceae bacterium 5_1_57FAA**	0.191737769
**Clostridiales bacterium 1_7_47FAA**	0.116475801
**Streptococcus anginosus**	−0.064872198
**Clostridium sp. HGF2**	0.584390133
**Anaerotruncus colihominis**	0.311595789
**Bacteroides sp. 3_2_5**	0.072721578
**Clostridium leptum**	0.483691517
**Alistipes shahii**	0.072167705
**Bacteroides sp. 2_1_16**	0.085731573
**Clostridium symbiosum**	0.59242247
**Bacteroides sp. 2_1_56FAA**	0.082399653
**Lachnospiraceae bacterium 3_1_57FAA_CT1**	−0.046512506
**Streptococcus thermophilus**	0.1749352
**Bacteroides sp. D20**	0.22844312
**Bacteroides fragilis**	−0.008040424
**Clostridium bolteae**	0.581966175

The significantly increased pathway (M00338) post-surgery and its correlation with significantly changed bacteria at species level. The KEGG ID and bacterial names were listed, and the r value of Spearman correlation tests were as shown.

## Discussion

TAAD patients are at high risk for GICs after surgery; this, in turn, affects their recovery. Previous studies have suggested that the intestinal microbiota plays an important role in maintaining human environmental homeostasis and intestinal barrier functions, regulating the intestinal immune system and impacting nutrient absorption and energy metabolism^15,16^. Therefore, we believe that postoperative complications of the digestive system are intrinsically connected with changes in the structure, diversity, functional genes, and metabolic pathways of the intestinal microbiota.

We found that the levels of some types of bacteria, such as *Oscillibacter*, *Anaerotruncus*, *Alistipes*, and *Clostridium difficile*, were higher after surgery, many of which are associated with several diseases. For example, *Oscillibacter*, *Alistipes*, and *Clostridiales* are associated with cancer, metabolic diseases, aging, and cardiovascular disease, mainly by influencing the mechanistic target of the rapamycin (mTOR) signaling pathway^10,16–18^. *Alistipes* and *Clostridium difficile* have always been considered bacterial pathogens^19,20^. Alexander *et al*. found that *Alistipes* induces intestinal inflammation by taking advantage of the gut poison secreted by intestinal bacteria^21^. Research has also shown that *Clostridium difficile* infection is the leading cause of nosocomial diarrhea in the United States and has surpassed the infection rate of other health care-associated infections such as methicillin-resistant *Staphylococcus aureus* ^22,23^. Meanwhile, *Eubacterium* and butyrate-producing bacteria have become significantly less common. These bacteria are associated with the inhibition of inflammatory cytokine release, maintaining the integrity of the intestinal mucosal barrier and improving the function of the intestinal immune system^24,25^. These results demonstrate that the structure of the gut flora is significantly changed after surgery, and such variation may be associated with GICs after surgery.

Changes in the structure of the intestinal microbiota caused changes in the abundance of related functional genes and pathways. Genes related to the branched-chain amino acid (BCAA) transport system were higher in postoperative patients. Interestingly, enrichment of the BCAA transport system has also been observed in the gut microbiota of type 2 diabetes (T2D) patients^26^. However, the reason for this is unclear. Genes encoding flagellin, the monomeric protein of bacterial flagellum, are also higher in postoperative patients, and many experiments have shown that it causes inflammation in the intestine by binding to toll-like receptors^27–30^. We also found that tryptophan synthesis-related functional genes were less abundant in patients after surgery. Tryptophan is an essential and important functional amino acid. Its metabolite, 3-indolepropionic acid in intestinal, regulates the expression of tight junction proteins and modulates the expression of pro- and anti-inflammatory genes in intestinal epithelial cells^31–33^. Moreover, a lack of tryptophan is associated with various diseases^34–38^. Thus the gut microbiota synthesized tryptophan may be reduced after surgery, and subsequently caused GIC.

For the functional pathways, we found that genes involved in tetracycline resistance, lipopolysaccharide biosynthesis, and cobalamin biosynthesis were less abundant after surgery, while those involved in cysteine biosynthesis and many energy metabolism pathways showed an increasing trend. Further analysis of the potential correlations between these significantly altered functional pathways and intestinal bacteria in postoperative patients revealed that, only cysteine biosynthesis is positively correlated with increased microbiota after surgery. Cysteine is synthesized from homocysteine, and high level of homocysteine in the plasma (known as hyperhomocysteinemia) is an indicator of renal damage, hypertension, and cardiovascular disease^39–42^. Previous studies have shown that homocysteine can cause inflammation of vascular endothelial cells and is related to the pathogenesis of AD and abdominal aortic aneurysms^43,44^. An increase in cysteine synthesis after surgery may cause a decrease in homocysteine level; therefore, we hypothesize that the low level of homocysteine may reduce inflammation in vascular endothelial cells and significantly control blood pressure levels in postoperative patients. However, we did not find a significant reduction in homocysteine levels in the plasma of postoperative patients in HPLC analyses (data not shown). We believe that a larger number of patients should be examined to test our hypothesis.

WBC and NE levels were significantly higher after surgery, proving the existence of an inflammatory response in postoperative patients with GICs. We checked medical records of another 4 Type A AD patients, their WBC levels before and after surgery were 6.21 ± 1.08 and 13.52 ± 6.77 respectively (gram per liter blood), and their NE levels were 3.92 ± 1.19 pre-surgery and 11.4 ± 6.27 post-surgery. Although WBC and NE levels were also increased in non-GICs group, the statistic differences were not significant (p = 0.077 in t test for comparing WBC levels pre-surgery and post-surgery; p = 0.058 in t test for comparing NE levels pre-surgery and post-surgery), and the levels in non-GICs group were lower than in GICs group both before and after operation. Thus, the inflammation was more severe in GICs patients. As mentioned above, the changes in bacterial species, functional genes, and pathways could induce systemic and intestinal local inflammation; therefore, we believe that the reaction and development of the inflammatory response may be related to changes in the microbiota. ELISA results of inflammatory cytokines in plasma suggest that IL-2, IL-6, IL-10, and IL-8 have a tendency to increase after surgery, although the differences between pre-surgery and post-surgery groups were not significant (probably due to the small number of samples). We also found ALT and AST levels were increased after surgery, which indicated liver injury after surgery. Since previous studies found that the changes in microbiota structure were associated with several kinds of liver diseases, we believe that ALT and AST changes may also be caused by modification in microbiota structure.

We also found that other functional genes and pathways, such as the NarL family, spermidine/putrescine transport system permease proteins, nitroreductase, putrescine transport system permease proteins, and others, changed after surgery. Previous studies have shown that spermidine and putrescine are associated with cell growth, proliferation, and protein degradation^45^; however, the effects of their changes on preoperative and postoperative thoracic AD patients are not clear.

Thoracic AD is an acute pathological process of large blood vessels, and it has attracted extensive interest^46^ for it is characterized by dangerous pathogenic conditions and high fatality. The success rate of the operation and the occurrence of postoperative complications are deciding factors of patient mortality. Our study, as preliminary exploratory research, indicates that the structure of the intestinal flora significantly changes in postoperative patients with GICs. We consider that these changes have certain relationships with inflammation and liver function damage. Our research opens up new possibilities of treatment for patients suffering from abdominal complications after surgery. Large-scale sampling and sequencing are needed for a deeper understanding of the role of gut microbiota in the postoperative development of GICs in AD patients.

## Materials and Methods

### Patient recruitment

In all, 40 patients who were admitted to the Beijing Anzhen Hospital Cardiac Surgery Center from May 2015 to January 2016 and underwent thoracic AD surgery were enrolled in accordance with inclusion and exclusion criteria; 10 healthy volunteers from the Health Examination Center were also included. None of the patients or healthy volunteers suffered from a mental illness or digestive tract diseases, such as inflammatory bowel disease, irritable bowel syndrome, peptic ulcers, digestive tract tumors, hepatitis, liver cirrhosis, diabetes, obesity, and coronary heart disease. Patients and healthy volunteers were excluded if they had received antibiotics within the last month before recruitment.

Written informed consent was obtained from all participants. The study was approved by the Ethics Committee of Anzhen Hospital, and was carried out in accordance with the approved guidelines.

### Clinical tests and treatments

Clinical data, such as age, sex, and AD type, were collected from the Clinical Data Center of Anzhen Hospital. All patients were subjected to routine blood and biochemical examinations before and after surgery, including determination of the levels of white blood cells (WBCs), neutrophile granulocytes (NEs), alanine aminotransferase (ALT), aspartate aminotransferase (AST) and serum creatinine (sCr) by the clinical laboratory at Anzhen Hospital.

All enrolled patients were Stanford Type A AD, and underwent Bentall + Sun’s Procedure surgical intervention. They took prophylactic antibiotics one time before surgery, and took antibiotics continuously after surgery until their hemogram returned to normal level. Most of them were given Cefamandole (1 gram per 8 hours), and a few of them were given Cefuroxime (0.75 gram or 1 gram per 8 hours).

### Fecal sample collection and DNA extraction

Fresh fecal samples were collected within 24 h of cardiac surgery and before the prophylactic pre-operative antibiotics administration (control group). After surgery, the first fecal samples were collected from patients with GICs (usually 2~5 days after surgery) (experimental group). Samples qualified for sequencing were obtained from 14 patients in the control group and another 8 patients in the experimental group. Freshly collected stool samples (2 to 5 g) were immediately placed into a sterile sampling box, transferred by ice bath, and maintained at −80 °C until use.

The fecal DNA of each sample was extracted and purified from 300 mg feces using the StoolGen DNA Kit (CW2092, Beijing Cowin Bioscience Co., Ltd.). DNA concentration and quality were determined on a NanoDrop spectrophotometer. A DNA sample with a concentration greater than 15 ng/µL and a 260/280 value between 1.8 and 2.0 was considered to be a qualified DNA sample. Agarose gel electrophoresis was performed to identify DNA samples without degradation.

### Metagenome sequencing and data processing

A DNA sequencing library was constructed according to the manufacturer’s instructions (Illumina HiSeq X Ten System, Illumina, San Diego, California, U.S.A). Paired-end libraries with an insert length of approximately 350 bp were built and sequenced from both ends with a read length of 150 bp. The raw reads were filtered by removing adaptor sequences, low-quality reads, and host genome sequence contamination. Ilumina sequence data reported in the paper is provided on SRA database (SRP102260). Detailed microbiota data are shown in the Supplementary Tables S1 to S4.

### Bioinformatics analysis

MetaPhlAn (v2.0)^47^ was used to determine the relative abundance of bacterial species present in all samples. Alpha diversity was evaluated using Simpson’s diversity index, and the samples were clustered and illustrated by principal component analysis to show beta diversity. SOAPdenovo^48^ and MetaGeneMark^49^ were used to perform de novo assembly and gene prediction, respectively, with high-quality reads. All predicted genes were aligned using CD-HIT (identity > 95% and coverage > 90%)^50^ to construct a non-redundant gene catalog. To obtain the relative abundance of each gene, the high-quality reads from each sample were aligned against the gene catalog using SOAP2 (identity > 95%). Putative amino acid sequences from the gene catalog were aligned against KEGG databases (release 59.0) using BLASTP (e-value ≤ 1e-5).

### Detection of inflammatory cytokines

Inflammatory cytokines, including interleukin (IL)-2, IL-6, IL-8, and IL-10, were detected in the plasma using enzyme-linked immunosorbent assay (ELISA) kits (Ray Biotech Company, catalog numbers ELH-IL-2, ELH-IL-6, ELH-IL-8, and ELH-IL-10, respectively) according to the manufacturer’s instructions. Briefly, for each test, serum samples were diluted according to the regular range of the target cytokine in humans. The diluted samples, as well as standard protein solutions with gradient concentrations, were added in duplicate into 96-well plates pre-coated with the appropriate antibody for each target cytokine in each well (100 μL to each well) and incubated for 2.5 h at room temperature with gentle shaking. The solutions were discarded, and each well was washed four times with 1X Wash Solution and incubated with 100 μL 1X prepared biotinylated antibody for 1 h at room temperature with gentle shaking. The solution was discarded, and each well was washed four times and incubated with 100 μL prepared streptavidin solution for 45 min at room temperature with gentle shaking. The solution was discarded, and each well was washed four times and incubated with 100 μL TMB One-Step Substrate Reagent for 30 min at room temperature in the dark with gentle shaking. Stop Solution (50 μL) was added and absorbance was immediately read at 450 nm. The cytokine concentration was determined by linear regression to the standard curve.

### Statistical analysis

Cytokine concentration, species abundance, and gene/pathway abundance comparisons were made using the unpaired Student’s t-test. Correlations between species and genes/pathways were decided based on Spearman’s rank correlation tests. P < 0.05 was taken to indicate statistical significance for each test. All analyses were carried out using GraphPad Prism version 5.0 for Windows (GraphPad). A heatmap was constructed using HemI software (HemI version 1.0) (http://dx.doi.org/10.1371/journal.pone.0111988).

## Electronic supplementary material

Supplementary Information

Supplementary Dataset
