# Supplementary material for: Clinical Parameters and Gut Microbiome Changes Before and After Surgery in Thoracic Aortic Dissection in Patients with Gastrointestinal Complications
Source: Sci Rep. 2017 Nov 9;7:15228. doi: 10.1038/s41598-017-15079-0 (PMC5680333; doi:10.1038/s41598-017-15079-0)
Supplement: Supplementary file 1 — Supplementary Information [file 41598_2017_15079_MOESM1_ESM.doc]

**Supplementary data for:**

**Clinical Parameters and Gut Microbiome Changes Before and After Surgery in Thoracic Aortic Dissection in Patients with Gastrointestinal Complications**

Shuai Zheng^1,2,3^†, Shulin Shao^4^†, Zhiyu Qiao, Xue Chen^4^, Chunmei Piao^1,2,3^, Ying Yu^4^, Feng Gao^4^, Jie Zhang^4*^ & Jie Du^1,2,3*^

^1^ Beijing Anzhen Hospital, Capital Medical University, Beijing 100029, China.

^2^ Beijing Collaborative Innovation Centre for Cardiovascular Disorders, Beijing 100029, China.

^3^ The Key Laboratory of Remodeling-Related Cardiovascular Diseases, Ministry of Education, Beijing Institute of Heart Lung and Blood Vessel Diseases, Beijing 100029, China.

^4^ Department of Gastroenterology, Beijing Anzhen Hospital, Capital Medical University, Beijing 100029, China.

^5^ Department of Cardiovascular Surgery, Beijing Aortic Disease Centre, Beijing Anzhen Hospital, Capital Medical University, Beijing Institute of Heart Lung and Blood Vessel Diseases, Beijing Engineering Research Centre for Vascular Prostheses, Beijing 100029, China.

† These authors contributed equally to this work

* Correspondence and requests for materials should be addressed to: J.Z. ([zhangjie4155@sina.com](mailto:zhangjie4155@sina.com)) and J.D. (email: [jdu@bcm.edu](mailto:jdu@bcm.edu))

**Supplemental Table Legend**

**Supplemental Table 1. The bacterial content at genus level in pre- and post-operative thoracic aortic dissection patients**

The strains in genus levels and their relative content (ratio to the total microbiota counts) in each sample are shown in the supplemental table 1. The samples under “Pre-operation” title were from pre-operative patients, and the samples under “Post-operation” title were from post-operative patients.

**Supplemental Table 2. The bacterial content at species level in pre- and post-operative thoracic aortic dissection patients**

The strains in species levels and their relative content (ratio to the total microbiota counts) in each sample are shown in the supplemental table 2. The sample under “Pre-operation” title were from pre-operative patients, and the samples under “Post-operation” title were from post-operative patients.

**Supplemental Table 3. The functional gene content in pre- and post-operative thoracic aortic dissection patients**

The content of functional genes (ratio to the total metagenomic counts) in each sample are shown in the supplemental table 3. The KEGG ID of each gene are listed on the left column. The sample under “Pre-operation” title were from pre-operative patients, and the samples under “Post-operation” title were from post-operative patients.

**Supplemental Table 4. The functional pathway content in pre- and post-operative thoracic aortic dissection patients**

The content of functional pathways (ratio to the total pathway counts) in each sample are shown in the supplemental table 4. The KEGG ID of each pathway are listed on the left column. The sample under “Pre-operation” title were from pre-operative patients, and the samples under “Post-operation” title were from post-operative patients.
